# A Series of Photosensitizes for Fe^3+^ in Aqueous Solution and Cells With Colorimetric and Fluorescent Channels: Synthesis and Performance

**DOI:** 10.3389/fchem.2022.867808

**Published:** 2022-03-31

**Authors:** Ming Xu, Jiang-Tao Liao, Gang Chen, Yin-Yun Chen, Dan Liu, Li-Le Wang

**Affiliations:** ^1^ Institute of Translational Medicine, Hunan Provincial People’s Hospital/The First Affiliated Hospital of Hunan Normal University, Changsha, China; ^2^ Department of Gastroenterology Medicine, Hunan Provincial People’s Hospital/The First Affiliated Hospital of Hunan Normal University, Changsha, China; ^3^ Department of Respiratory Medicine, Hunan Provincial People’s Hospital/The First Affiliated Hospital of Hunan Normal University, Changsha, China

**Keywords:** fluorescent imaging, rhodamine chemosensor, fe 3+ imaging, colormetric sensing, luminescence

## Abstract

Ferrum (Fe) is a widely existing metal element and nearly the most important trace element in living species, including human beings. The design of chemosensors for Fe ions faces issues related to the d-d transition of Fe(II) and Fe(III) ions, which makes them efficient electron trappers and energy quenchers. Most fluorescent dyes cannot afford such d-d quenching, showing emission turn off effect towards both Fe(II) and Fe(III) ions with poor selectivity. As a consequence, the development for Fe with emission turn on effect and good selectivity shall be continued and updated. In this work, three rhodamine-derived chemosensors modified by different lengths of alkyl chains having electron-donating N and O atoms were synthesized and explored for the selective optical sensing of Fe(III). These chemosensors showed colorimetric and fluorescent emission turn on sensing for Fe(III), showing two sensing channels. These chemosensors showed good selectivity, which was assigned to the sieving effect of alkyl chains with electron-donating N and O atoms. The N atom was found to be more effective in associating with Fe(III), compared to the O atom. Their fluorescent cell imaging experiment was carried out to confirm the possibility of being used for cell imaging.

## Introduction

Ferrum (Fe) is a widely existing metal element and one of the most important trace elements in living species, including human beings. Most crucial biological activities require the participation of Fe, such as the transportation of O_2_ by hemoglobin in red blood cells (Liu et al., 2002; Tumambac et al., 2004; Xiang and Tong, 2006). An abnormal level of Fe in the human body or heavy daily Fe intake can cause disorder of vital organs, such as the kidney, liver, bowel, and even brain. More seriously, some cancers have been associated with some serious diseases including leukocythemia and hematosepsis (Bricks et al., 2005; Ghosh et al., 2009; Mitra et al., 2009). Fe accumulation and pollution bring harmful effects to the ecosystem, which makes Fe concentration an indicator of the health of local ecosystems. Regardless of its importance at the cellular level in the human body as well as environmental protection, photosensitizers for optical sensing, whether colorimetric or fluorescent, are not yet well understood. The design of chemosensors for Fe ions has issues related to the fact that the d-d transition of Fe(II) and Fe(III) ions makes them efficient electron trappers and energy quenchers. Nearly all fluorescent dyes cannot afford such d-d quenching, showing an emission turn off effect towards both Fe(II) and Fe(III) ions with poor selectivity. As a consequence, the development for Fe with emission turn on effect and good selectivity requires further research.

There is a simple signaling method of colorimetric sensing based on the color or absorbance intensity variation triggered by the analyte (Gedye et al., 1986). A representative example of colorimetric sensing is naked eye detection, which, however, suffers from limited precision. In other words, colorimetric sensing should be assisted by other methods. There is another simple method of fluorescent sensing, which has been widely used for both *in vitro* and *in vivo* signaling. Its advantages include high sensitivity, unique selectivity, instant response, simple pretreatment, non-invasive detection, and so on, which can be applied for real-time in-site monitoring and *in vitro*/vivo sensing (Beeri et al., 1986; Bühler and Feldmann, 2006; Hu et al., 2007; Vargas-Hernandez et al., 2010; Yuan et al., 2012). Thus, the combination of colorimetric and fluorescent signaling is an ideal pathway to develop the desired chemosensors of Fe with *in vitro*/vivo sensing properties.

As a class of excellent chemosensors/photosensitizers for metal cations and small biomolecules, rhodamine and its derivatives are well-known for their structural transformation between spirolactam and ringing-open structures, which is accompanied by simultaneous colorimetric and fluorescent sensing. Most rhodamine molecules have been reported as promising chemosensors by properly constructing a suitable coordination site for the desired species/analyte (He et al., 2011; Yan et al., 2012; Betzig, 2015). In a typical sensing mechanism, as-synthesized rhodamine molecules take their energy-favored formation, which is both colorless and non-fluorescent in the visible region. Given a specific analyte, these rhodamine molecules coordinate with it and transform into an open-ring formation which is both colorful and highly fluorescent in the visible region. Such structural transformation can be modified and finely controlled to serve optical sensing for heavy metal ions, even though there is still a strong d-d quenching effect on rhodamine emission. As a result, fluoresce turn on chemosensors for heavy metals such as Hg(II), Cu(II) and Pb(II) have been realized (Buffat et al., 1991; Tanaka et al., 2003; He et al., 2011; Yan et al., 2012; Betzig, 2015).

**SCHEME 1:**
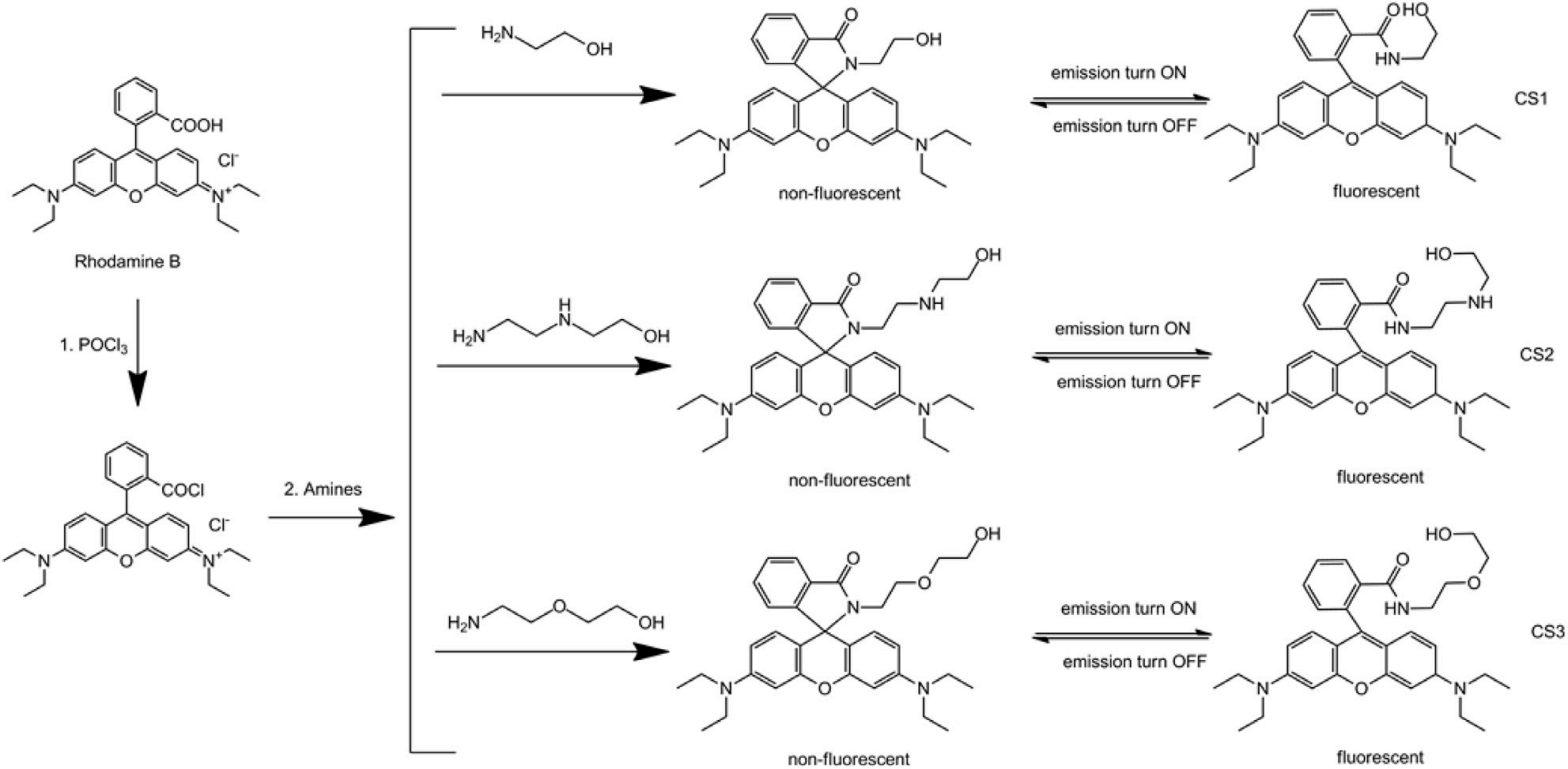
Synthetic protocol for CS1-3.

Based on these past studies, it is assumed that chemosensors for Fe(II) and Fe(III) ions with good selectivity will be constructed using a neat molecular design that offers a fixed coordination site for only Fe(II) or Fe(III), so that good selectivity, along with colorimetric and fluorescent sensing, can be obtained. In this work, we modified rhodamine B molecules with different lengths of amine and alcohol chains to find suitable coordination sites for Fe(II) or Fe(III) ions. The corresponding synthetic route is shown in Scheme 1. Their colorimetric and fluorescent sensing behavior in aqueous cells is also explored and discussed.

## Experimental Details

### Materials and Instruments

The compounds and reagents used in this work were all analytical grade. Organic solvents and water for this work were double-distilled and treated with an N_2_ stream to exclude dissolved O_2_. Metal nitrate salts were commercially obtained from Tianjin Chemical Company. Starting compounds, including rhodamine B, POCl_3_, 2-hydroxyethylamine, N-(2-aminoethyl)ethanolamine, and O-(2-hydroxyethyl)ethanolamine, were gained from Shiyan Chemical Company. Fluorescence spectra were collected by a Hitachi F-7000 fluorescence spectrophotometer, with an excitation source of a Xe lamp (150 W). A quartz cuvette (optical length = 10 mm) was applied, with excitation and emission bandpasses of 5 nm. Absorption features were determined by a Shimadzu UV-3000 spectrophotometer. NMR spectra were recorded with a Mercury-300BB spectrometer (Varian), where tetramethylsilane (TMS) was added as an internal standard. Single crystal data were collected by a Bruker Smart Apex CCD single crystal diffractometer. The above experiments were carried out in ambient conditions without being specified.

### Synthetic Operation of CS1-3

Chemosensor CS1: rhodamine B powder (10 mmol) was mixed with CH_2_Cl_2_ (20 ml). POCl_3_ was slowly mixed with this solution and stirred at 60°C for 8 h. Solvent and POCl_3_ residue were then extracted by rotary evaporation. Anhydrous acetonitrile was added to a solid product and stirred for 20 min. Then 2-hydroxyethylamine (1 ml, excess) was added into the solution. The resulting mixture was heated at 80°C for 8 h under N_2_ atmosphere (Shang et al., 2012). After natural cooling, the solid product was collected and purified by silica gel column with CH_2_Cl_2_:*n-*hexane (1:20) as eluent. Pink solid was obtained as CS1 with a yield of 59%. ^1^H NMR (CDCl_3_) δ 8.01 (s, 1H), 7.34–7.31 (m, 3H), 6.71 (s, 2H), 6.34 (s, 4H), 3.93–3.87 (m, 4H), 3.63–3.50 (m, 8H), 1.16 (t, 12H). ^13^C NMR δ 167.55, 152.14, 149.50, 145.22, 132.10, 130.62, 128.00, 125.99, 125.64, 122.41, 115.94, 110.17, 102.05, 65.51, 60.14, 44.51, 43.36, 12.37. Elemental analysis for C_30_H_35_N_3_O_3_, was calculated C: 74.20, N: 8.65, H: 7.26, and obtained C: 74.13, N: 8.71, H: 7.34.

For Chemosensor CS2, its synthetic procedure is similar to that of CS1, except that 2-hydroxyethylamine was replaced by N-(2-Aminoethyl)ethanolamine (2 ml, excess) in this work. Pink solid was obtained with a yield of 47%. ^1^H NMR (CDCl_3_) δ 8.00 (s, 1H), 7.33–7.30 (m, 3H), 6.70 (s, 2H), 6.35 (s, 4H), 3.84–3.77 (m, 4H), 3.53–3.50 (m, 8H), 3.12 (bs, 1H), 2.92–2.90 (m, 4H), 1.15 (t, 12H). ^13^C NMR δ 167.33, 152.12, 149.49, 145.43, 132.11, 130.61, 128.01, 125.97, 125.63, 122.40, 115.87, 110.19, 102.06, 65.54, 60.65, 50.82, 47.92, 44.50, 44.02, 12.36. Elemental analysis for C_32_H_40_N_4_O_3_, was calculated C: 72.70, N: 10.60, H: 7.63, and obtained C: 72.66, N: 10.69, H: 7.78.

For Chemosensor CS3 the synthetic procedure is similar to that of CS1, except that 2-hydroxyethylamine were replaced by O-(2-Hydroxyethyl)ethanolamine (2 ml, excess) in this work. Pink solid was obtained with a yield of 45%. ^1^H NMR (CDCl_3_) δ 7.98 (s, 1H), 7.33–7.31 (m, 3H), 6.72 (s, 2H), 6.34 (s, 4H), 3.97 (m, 2H), 3.72–3.69 (m, 4H), 3.53–3.50 (m, 10H), 1.15 (t, 12H). ^13^C NMR δ 167.37, 152.11, 149.50, 145.11, 132.10, 130.62, 128.00, 125.98, 125.62, 122.41, 115.71, 110.17, 102.05, 71.81, 69.01, 65.50, 61.45, 44.51, 41.88, 12.33. Elemental analysis for C_32_H_39_N_3_O_4_, was calculated C: 72.56, N: 7.93, H: 7.42, and obtained C: 72.45, N: 7.99, H: 7.53.

### Fe Ion Sensing Operation

Each chemosensor was dissolved in ethanol to form a stock solution (1 mM). For spectroscopic measurements, each stock solution was diluted with ethanol until the concentration was 1 × 10^–5^ mol L^−1^. As for the stock solutions of metal ions, corresponding metal nitrates were dissolved in distilled water and diluted to 1 mM for later use.

During Fe(III) ion sensing operation, each chemosensor stock solution was added into a 10-ml glass tube, target metal nitrate salts were finely weighed and added, then distilled water was added until the solution volume was fixed as 10 ml. After being shaken for 3 min, spectroscopic measurements were performed immediately.

### Cell Imaging

The cell imaging performance of these chemosensors was carried out with Rat Schwann cells (RSC 96). These cells were firstly cultured in H-DMEM (Dullbecco’s modified eagle’s medium, high glucose) supplemented with 10% FBS (fetal bovine serum). Then, these cells were cultured by each chemosensor (10 μM) for 10 min at 37°C. Finally, metal ion solution (50 μM) was poured into these cells before fluorescence imaging with an Olympus fluorescence microscope (BX51).

## Results and Discussion

### Design Strategy of CS1-3

As the mentioned above, rhodamine molecules are taking a stable spirolactam structure to favor low energy which is both non-fluorescent and colorless in the visible region. Given a suitable coordination center, such as a metal ion, these rhodamine molecules transform their structure to fit this coordination center, forming an open-ring structure that is both highly fluorescent and colorful in the visible region. As a consequence, two sensing channels, colorimetric and fluorescent sensing, can be realized. Generally, the design and exploration of a suitable coordination site in a rhodamine chemosensor is the key factor controlling sensitivity and selectivity. In this work, we modify a rhodamine B molecule with alkyl chains with different electron donators (N and O atoms). These alkyl chains with electron donators may form coordination sites for metal ions. The spectral response and sensing performance of these chemosensors are compared to find the optimal chemosensor and corresponding coordination site.

### Single Crystal Structure of CS1

**FIGURE 1:**
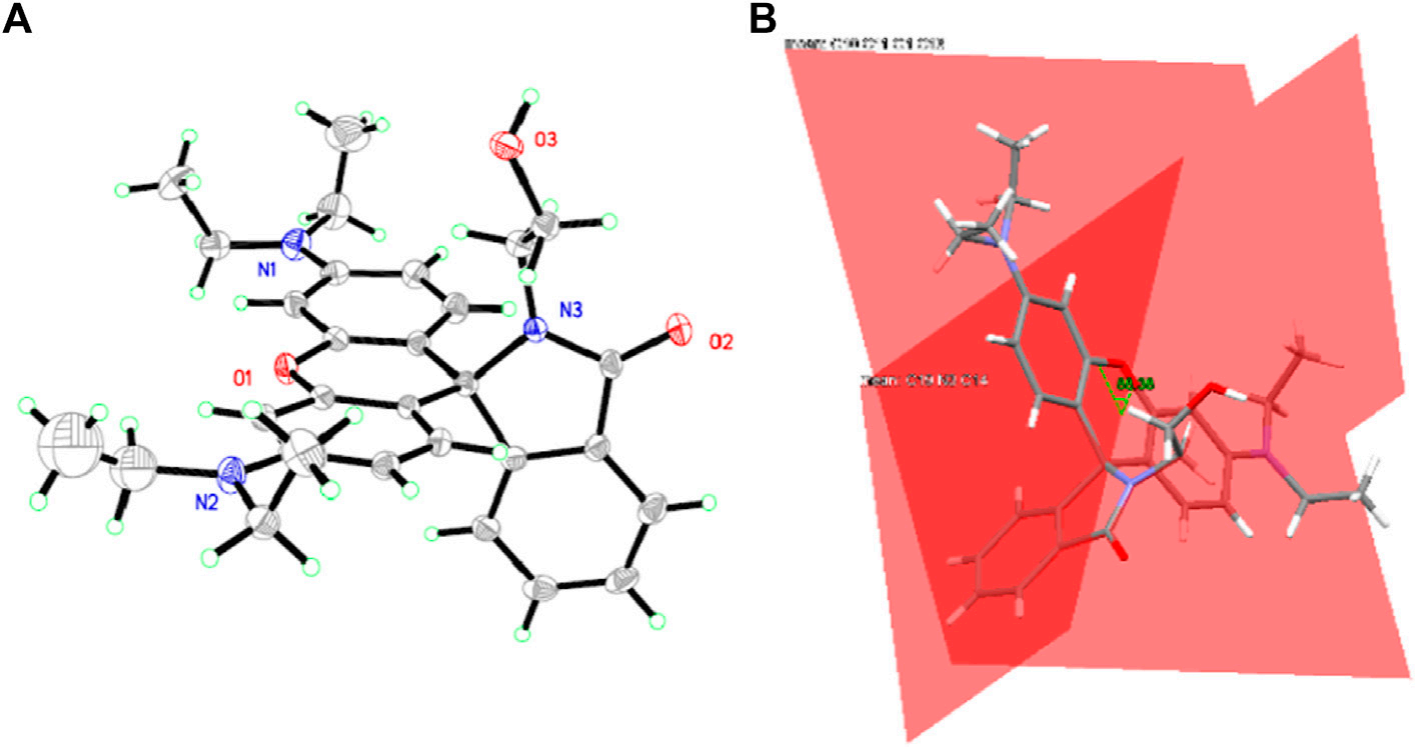
**(A)** single crystal structure of CS1. **(B)** the dihedral angle between spirolactam ring and heteroanthracene ring.

Due to the coplanar planes in CS1, its molecules tend to form an ordered alignment, resulting in a single crystal. The geometric structure of CS1 is shown in Figure 1. Detailed structural parameters are listed in the Supplementary Material. As for CS2 and CS3, their longer alkyl chains may compromise their rigidity, so that no single crystals are successfully obtained. It can be observed from Figure 1 that CS1 is taking a typical spirolactam structure. This is because this spirolactam structure is a thermally stable one. CS1 molecules crystalize in the triclinic system, with cell length values of 9.22 (a), 12.14 (b), and 14.89 Å (c), respectively, which are similar to literature reports (Zhang et al., 2014; Helmbrecht et al., 2015). The introduced alkyl chain is observed in CS1, which confirms its successful synthesis. The spirolactam ring is nearly vertical to the heteroanthracene ring with a dihedral angle of 88.38^o^, which stops the conjugation in CS1 and explains its non-fluorescent nature in the absence of a coordination center.

### Absorption Spectra of CS1-3 Upon Fe(III)

**FIGURE 2:**
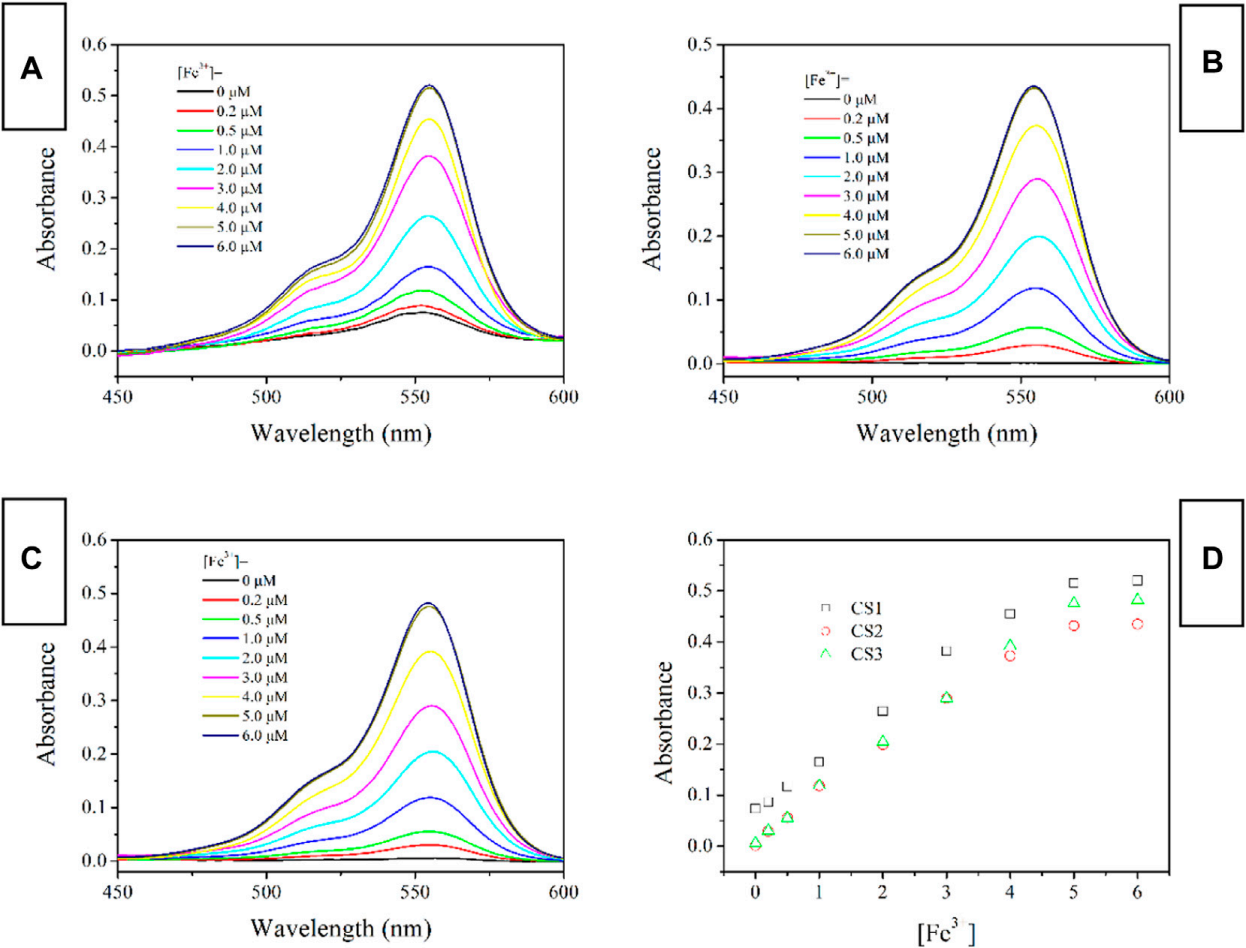
Absorption spectra of chemosensors (10 μM) with the addition of Fe^3+^ (0.0–6.0 equiv) in the aqueous (containing 5% ethanol as a co-solvent), **(A)** CS1; **(B)** CS2; **(C)**, CS3; **(D)**, absorbance variation at 555 nm of these chemosensors.

Considering the above assumption about two sensing channels, colorimetric and fluorescent sensing, the absorption response of these chemosensors CS1-3 is first revealed through their absorption spectra upon increasing Fe(III) concentrations [denoted as (Fe^3+^)]. As shown in Figure 2, no obvious absorption in the visible region is detected for CS2 and CS3 in a mixed solvent of H_2_O/ethanol at neutral conditions (10 μM, pH = 7). This observation suggests that the as-synthesized CS2 and CS3 molecules tend to take their spirolactam formation in the ground state. As for CS1, a weak but detectable absorption at 555 nm is observed, indicating the partial CS1 molecules are taking their open-ring structure even in the ground state. This may be explained by the terminal–OH group which may form an H bond with the acid amide of rhodamine, leading to the ring opening. In CS2 and CS3, their alkyl chains are longer than that in CS1, making their–OH group far away from the acid amide of rhodamine, showing no ring opening. Upon increasing [Fe^3+^], an absorption band peaking around 555 nm is observed for all three chemosensors, which shall be attributed to the characteristic absorption of rhodamine open-ring structure (Zhang et al., 2014; Helmbrecht et al., 2015).

### Fluorescence Spectra of CS1-3 Upon Fe(III)

**FIGURE 3:**
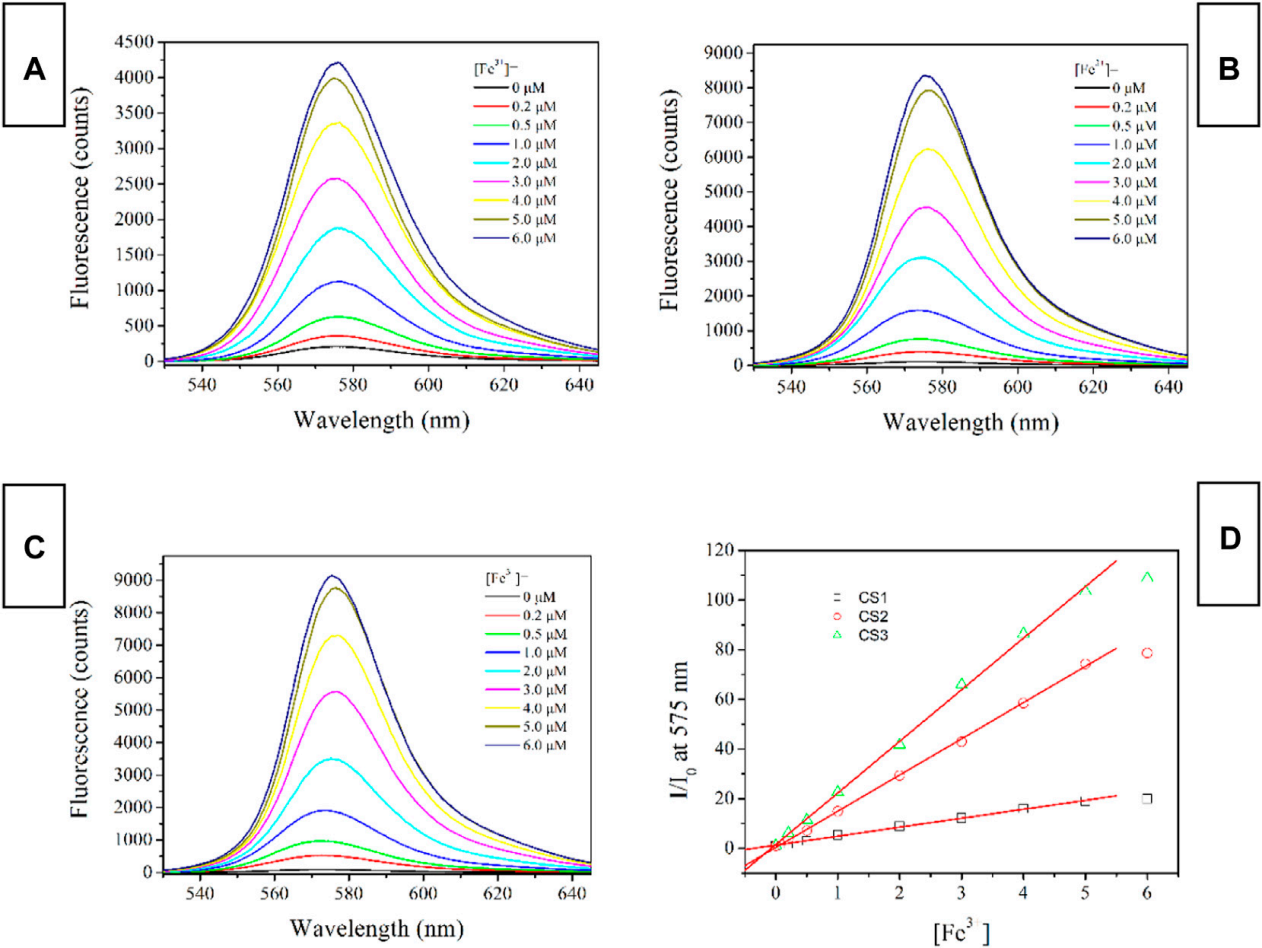
Emission spectra of chemosensors (10 μM) with the addition of Fe^3+^ (0.0–6.0 equiv) in the aqueous (containing 5% ethanol as a co-solvent), **(A)**, CS1; **(B)**, CS2; **(C)**, CS3; **(D)**, I/I_0_ variation at 575 nm of these chemosensors. Here I means fluorescence intensity and I_0_ is the fluorescence intensity without Fe^3+^.

The fluorescent spectra of CS1-3 upon increasing Fe(III) concentrations in a mixed solvent of H_2_O/ethanol at neutral condition (10 μM, pH = 7) are shown in Figure 3 (excitation wavelength = 500 nm). When (Fe^3+^) is as low as 0 μM, all three CS chemosensors show weak emission signals in the visible region, which means that most of these CS1-3 molecules are taking their spirolactam formation in the ground state. This observation is consistent with the above result of absorption. Corresponding emission quantum yields are determined as 0.03 for CS1, <0.01 for CS2 and <0.01 for CS3, respectively. Given a small portion of Fe(III), an obvious fluorescence enhancement is observed, peaking at ∼575 nm. A gradual fluorescent intensity enhancement is observed with increasing [Fe^3+^], showing an emission turn on effect. Such enhanced fluorescence has been attributed to the generation of rhodamine ring-opened amide form which is transformed from rhodamine spirolactam form caused by metal cations (Zhang et al., 2014; Helmbrecht et al., 2015). No obvious spectral shifts or new peaks are observed, suggesting that the emission turn on effect triggered by Fe(III) is stable. It appears that CS1 suffers from a limited fluorescence enhancement, compared to CS2 and CS3 given the same [Fe^3+^]. This is because, as mentioned above, some CS1 molecules are taking spirolactam formation in the ground state, meaning that CS1 shows weak but detectable fluorescence in the absence of Fe(III). As for CS2 and CS3, no obvious fluorescence is observed in the absence of Fe(III). As a consequence, even though CS1-3 all exhibit emission turn on effects triggered by Fe(III), the fluorescent enhancement of CS1 is always lower than those of CS2 and CS3. At 5 equivalent of [Fe^3+^], CS1 fluorescence becomes 19.9-fold higher than its original value ([Fe^3+^] = 0 μM), while, CS2 and CS3 fluorescence intensities are enhanced by 78.7-fold and 108.7-fold, respectively, upon 5 equivalent of [Fe^3+^]. The corresponding emission quantum yields were determined as 0.59 for CS1, 0.78 for CS2, and 0.84 for CS3, respectively. Given a higher [Fe^3+^] of 6 equivalent, the fluorescent intensity of CS1-3 is only slightly increased, suggesting that all CS molecules have been associated with Fe(III) ions. With a complete structural transformation from spirolactam form to ring-opened amide form, CS fluorescent intensity is increased no more.

### Selectivity of CS1-3 Upon Fe(III)

**FIGURE 4:**
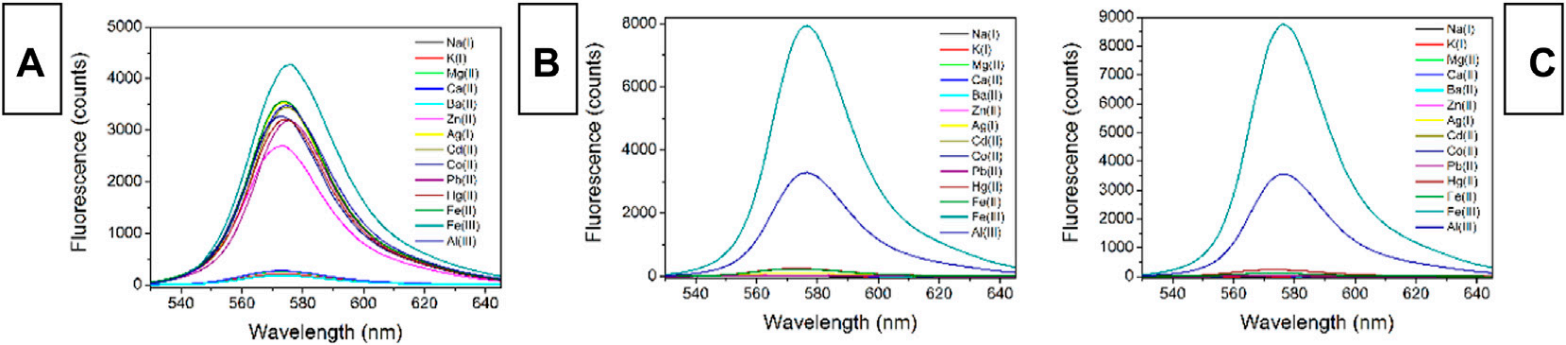
Emission spectra of chemosensors (10 μM) upon various interfering metal ions (5.0 equiv) in the aqueous (containing 5% ethanol as a co-solvent), **(A)**, CS1; **(B)**, CS2; **(C)**, CS3.

To examine sensing selectivity of CS1-3 for Fe(III) over competing metal ions, their fluorescent spectra upon the presence of common metal cations were recorded, as shown in Figure 4. For all three CS chemosensors, alkaline metal and alkaline earth metal cations (Na^+^, K^+^, Mg^2+^, Ca^2+^, Ba^2+^) exert a slim effect on them, showing no obvious emission turn on effect, except for CS1’s instinct fluorescence residue mentioned above. These small metal cations fail to match the coordination sites in CS1-3. Given the transition metal cations (Zn^2+^, Ag^+^, Cd^2+^, Co^2+^, Pb^2+^, Hg^2+^, Fe^2+^), CS1 fluorescence enhancement is observed for nearly all these metal cations. Although the enhanced fluorescence intensity by these transition metal cations is not as effective as that by Fe(III), CS1 fluorescence is enhanced, indicating a poor selectivity of CS1. No similar enhanced fluorescence is observed for CS2 and CS3, however, when they are exposed to nitrate salts of alkaline and alkaline earth metal cations (Na^+^, K^+^, Mg^2+^, Ca^2+^, Ba^2+^), transition metal cations (Zn^2+^,Ag^+^, Cd^2+^, Co^2+^, Pb^2+^), and even Hg(II) and Fe(II) ions. A good selectivity for Fe(III) over competing metal cations is thus confirmed for CS2 and CS3. Considering the similar molecular structures between CS1-3, their selectivity difference was assigned to their different lengths of alkyl chains having N/O atoms. The alkyl chain of CS1 is short with an O atom at the end of this alkyl chain, meaning that its coordination site is available for most transition metal cations. In this case, poor selectivity is observed for CS1. In CS2 and CS3, their alkyl chains are prolonged, so that their coordination site forms a specific cage for Fe(III), which explains their unique selectivity towards Fe(III) (Xie et al., 2007; Chang et al., 2013). Further explanation of the selectivity of Fe(III) over Fe(II) assumed that these chemosensors have a strong affinity for Fe(III) due to their higher charge than Fe(II), leading to their spectral response for Fe(III). As for Fe(II), its lower affinity for chemosensors fails to trigger chemosensor structural transformation. This hypothesis is consistent with the observation of a higher interfering effect from Al(III), as shown in Figure 4. As for Hg(II), its atomic radius may mismatch the coordination site of these chemosensors, leading to its limited effect on selectivity.

**FIGURE 5:**
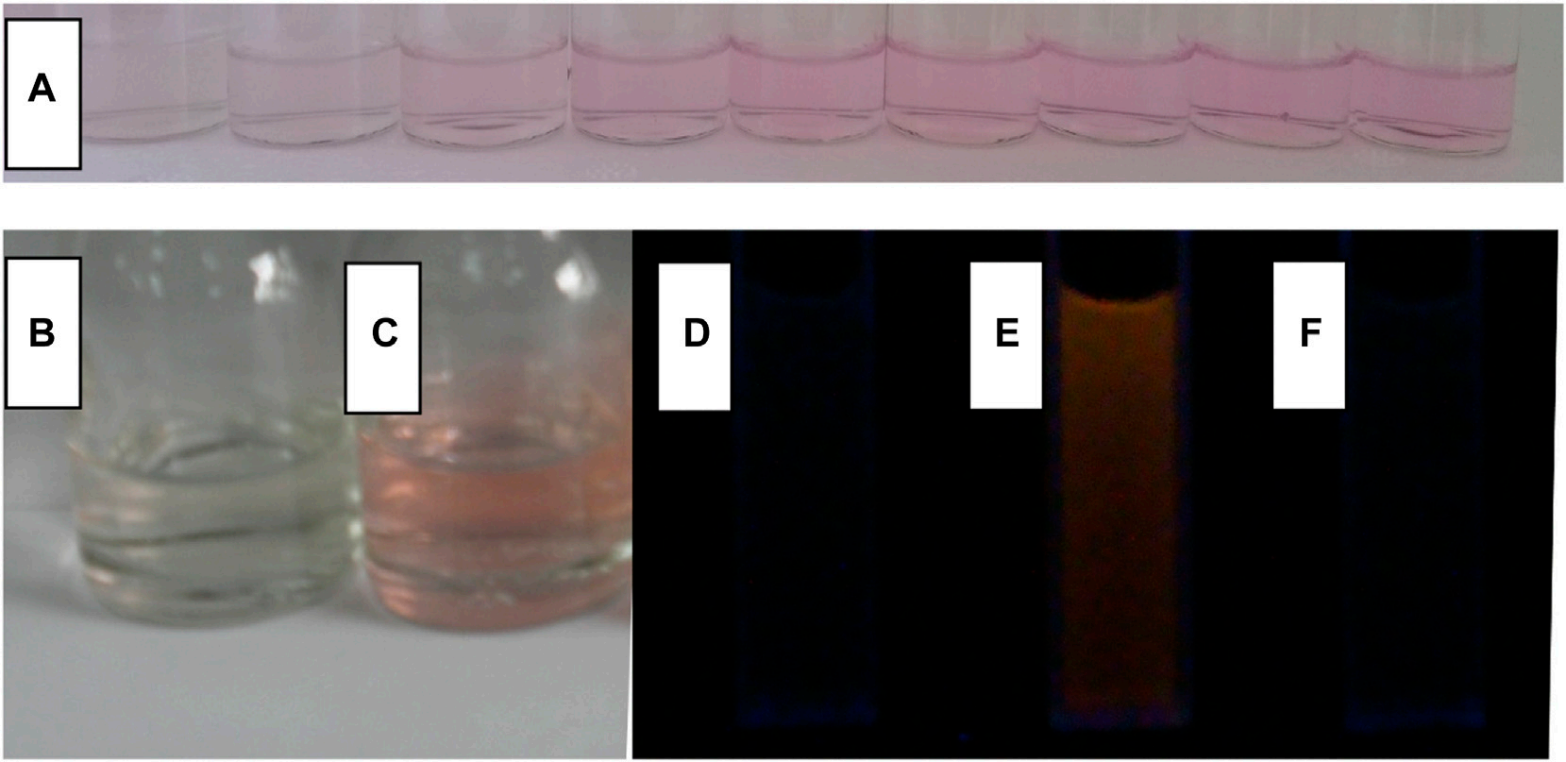
In the aqueous (containing 5% ethanol as a co-solvent), **(A)**, a photo of CS2 (10 μM) with the addition of Fe^3+^ (from left to right, 0, 0.2, 0.5, 1, 2, 3, 4, 5 and 6 μM); a photo of CS2 (10 μM) without **(B)** and with **(C)** Fe^3+^ (5.0 equiv), an emission photo of CS2 (10 μM) without Fe^3+^ **(D)**, with Fe^3+^ [5.0 equiv, **(E)**], and sequentially adding Fe^3+^ and EDTA [5.0 equiv:5.0 equiv, **(F)**].

Due to the good selectivity of CS2 and CS3, their solutions remain still colorless and transparent in the presence of these competing metal cations. After adding Fe(III), their color becomes pink, as shown in Figure 5, which confirms the possibility of naked eyes detection. Correspondingly, enhanced fluorescence is observed, even in the presence of these competing metal cations. It is thus confirmed that CS2 and CS3 have shown unique selectivity for Fe(III) in a complicated competing environment. As for CS1, its selectivity is compromised by its short alkyl chain, as above mentioned. The time-resolved experiment suggests that the complexation procedure between CS2 and Fe(III) can be finished within 600 s, including both colorimetric and fluorescent responses. While it will take CS3 much more time (>12 h) to finish the same complexation procedure with Fe(III). This rather different activity between CS2 and CS3 is attributed to the different coordination affinities of the N atom (in CS2) and O atom (in CS3). In other words, the N atom (CS2) has a stronger coordination affinity for Fe(III) than the O atom (CS3). With the addition of EDTA (5 equiv.) into the mixture of CS2 and Fe(III), the enhanced fluorescence is reversed back to its original intensity, indicating that the structural transformation between spirolactam form and ring-opened amide form is reversible under a suitable competing reagent. The instant sensing response of CS2 makes it a practical chemosensor for Fe(III) detection. While CS1 suffers from limited selectivity, CS3 shows rather slow response behavior, which compromises their further application.

### The pH Effect on CS1-3

**FIGURE 6:**
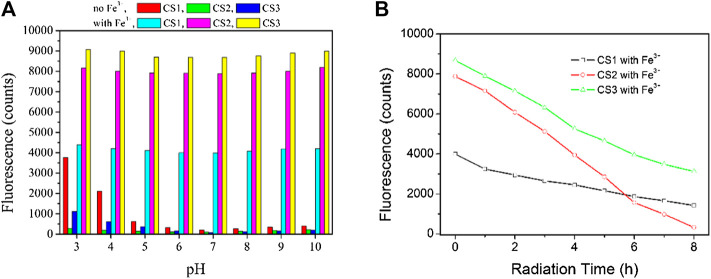
**(A)** Emission intensity variation of these chemosensors (10 μM) upon various pH values, without and with Fe^3+^ (5.0 equiv) in the aqueous (containing 5% ethanol as a co-solvent). **(B)** emission intensity monitoring of these chemosensors (10 μM) upon continuous radiation, with Fe^3+^ (5.0 equiv) in the aqueous (containing 5% ethanol as a co-solvent), excitation wavelength = 550 nm.

It has been reported that rhodamine molecules can initiate their structural transformation with the help of a proper coordination center, not only metal cation but also small molecules such as amino acids and even protons (Xie et al., 2009; Chang et al., 2013). Given the possibility of the structural transformation caused by protons, the pH effect on the sensing performance of CS1-3 will be evaluated. As depicted by Figure 6, CS1 is affected by pH values lower than 5. Even in the absence of Fe(III), CS1 fluorescence is increased by free protons in an aqueous solution. At pH = 3, CS1 fluorescence intensity is even close to the fluorescence intensity of CS1 with 5 equivalent of Fe(III). This observation is explained by the fact that rhodamine structural transformation can also be triggered by protons, similar to the coordination of Fe(III). As for CS2 and CS3, the pH effect on enhancing their fluorescence intensity is less obvious, indicating that most CS2 and CS3 molecules preserve their spirolactam form even in the presence of excess protons. Considering the rather similar molecular structures of CS1-3, their different responses towards pH shall be explained by their different lengths of alkyl chains with N/O atoms. It is assumed that the N atoms in CS2 and CS3 alkyl chains may act as a buffering site for excess protons, which weakens their effect on CS2/3 structural transformation (Xie et al., 2009). As for CS1, no such buffering N atoms can be offered. As a consequence, CS2 and CS3 show improved fluorescence stability under varying pH values. Although the pH effect on CS1-3 fluorescence is not neglectable, their fluorescent response towards Fe(III) is still preserved. By adding 5 equivalent of Fe(III), CS1-3 can enable enhanced fluorescence, regardless of the various pH values. This result suggests that CS1-3 prefers Fe(III) coordination instead of being neutralized with protons, due to Fe(III)’s higher coordination affinity (Xie et al., 2009; Wen et al., 2011). Upon even lower pH values (<3) or even higher ones (>10), all chemosensors fail to remain in their spirolactam form, showing obvious emission interference due to the strong interfering effect from excess protons or–OH ions. As a consequence, these chemosensors should be applied to a gentle environment, such as buffer solution, with a pH region of 3–10.

Photostability is another important parameter to evaluate chemosensor performance. Upon excitation wavelength of 550 nm, the emission intensity of these chemosensors is monitored in the presence of Fe^3+^ (5.0 equiv). It can be observed from Figure 6 that CS2 suffers from an obvious photobleaching effect, which can be explained by its multiple electron-donating N atoms, which are vulnerable to the oxidization effect.

### Fe^3+^ Imaging in Cells by CS1, CS2, and CS3

**FIGURE 7:**
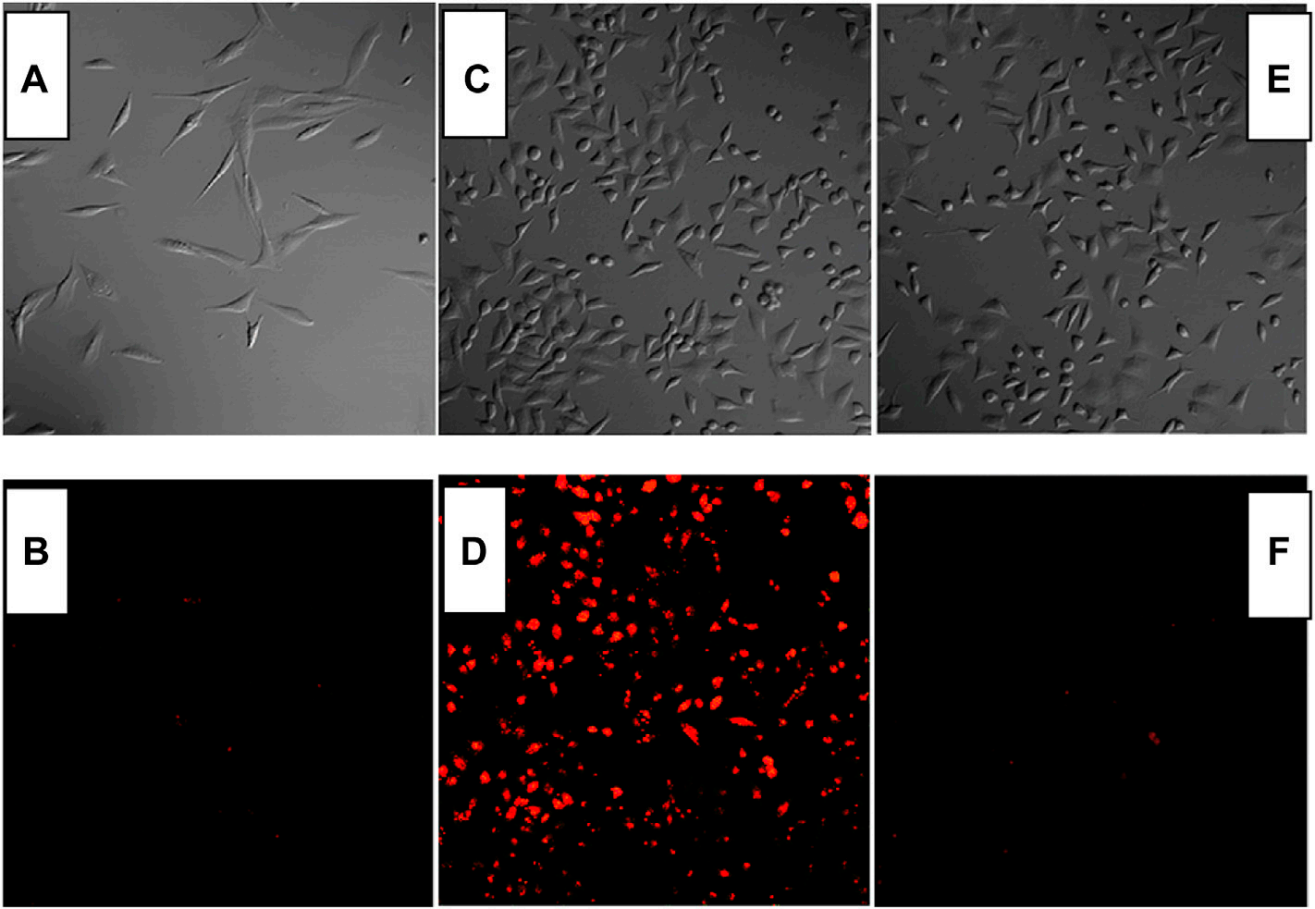
Fluorescent imaging of Rat Schwann cells stained by CS1 **(A,B)**, CS2 **(C,D)**, and CS3 [**(E,F)**, 10 μM] without and with Fe^3+^ (50 μM).

Combined with the above result, it is concluded that CS2 may serve for Fe(III) cell imaging by showing strong fluorescence, better selectivity, and fast response. As for CS1, its weak emission and limited selectivity hold from a further application, while CS3 needs a rather long reaction time with Fe(III), which compromises its practical application as well. To confirm this hypothesis, Fe(III) cell imaging is performed using CS1-3. As depicted by Figure 7, no obvious fluorescence signal was observed under a fluorescence microscope for the cells stained by only CS1-3. Even for CS1-stained cells, no obvious fluorescence was observed, which can be explained by its weak fluorescence. After being cultured by Fe(III), bright fluorescence was observed for CS2-stained cells, indicating that it can be used for cell imaging with good biocompatibility and permeability. The practical application of CS2 for Fe(III) cell imaging is hereby confirmed. As for CS1 and CS3, no bright fluorescence were observed, which can be attributed to the relatively weak fluorescence of CS1 and the long reaction/coordination time of CS3 with Fe(III).

## Conclusion

As a conclusion, this paper reported three chemosensors based on rhodamine B, various lengths of alkyl chains having electron-donating N and O atoms were grafted onto a typical rhodamine B molecule. A corresponding single crystal structure was obtained, which confirms the successful synthesis. Their absorption and fluorescence spectra upon Fe(III) and other competing metal cations were recorded and compared. It was found that CS1-3 showed two sensing channels for Fe(III) with good selectivity. Its colorimetric sensing showed increased absorbance with increasing Fe(III) concentration. Its fluorescence intensity showed turn on effect by Fe(III). The good selectivity of CS2 and CS3 was attributed to the sieving effect of alkyl chains having electron-donating N and O atoms. It was found that the N atom was more effective in associating with Fe(III), compared to the O atom. Their fluorescent cell imaging experiment was carried out to confirm the possibility of being used for cell imaging. For later experiments, efforts should be devoted to the improvement and modification of these chemosensors, aiming at better sensitivity.

## Data Availability

The datasets presented in this study can be found in online repositories. The names of the repository/repositories and accession number(s) can be found in the article/Supplementary Material.
