# Supplementary material for: A Series of Photosensitizes for Fe3+ in Aqueous Solution and Cells With Colorimetric and Fluorescent Channels: Synthesis and Performance
Source: Front Chem. 2022 Mar 31;10:867808. doi: 10.3389/fchem.2022.867808 (PMC9008735; doi:10.3389/fchem.2022.867808)
Supplement: Supplementary file 1 [file Table1.DOCX]

Supplementary Material

# Supplementary Data

data_4

_audit_creation_method SHELXL-97

_chemical_name_systematic

;

?

;

loop_

_atom_type_symbol

_atom_type_description

_atom_type_scat_dispersion_real

_atom_type_scat_dispersion_imag

_atom_type_scat_source

C C 0.0033 0.0016 'International Tables Vol C Tables 4.2.6.8 and 6.1.1.4'

H H 0.0000 0.0000 'International Tables Vol C Tables 4.2.6.8 and 6.1.1.4'

N N 0.0061 0.0033 'International Tables Vol C Tables 4.2.6.8 and 6.1.1.4'

O O 0.0106 0.0060 'International Tables Vol C Tables 4.2.6.8 and 6.1.1.4'

_symmetry_cell_setting Triclinic

_symmetry_space_group_name_H-M P-1

loop_

_symmetry_equiv_pos_as_xyz

'x, y, z'

'-x, -y, -z'

_cell_length_a 9.225(2)

_cell_length_b 12.144(3)

_cell_length_c 14.886(4)

_cell_angle_alpha 74.73(1)

_cell_angle_beta 78.29(2)

_cell_angle_gamma 83.99(4)

_cell_volume 1572.9(6)

_cell_formula_units_Z 2

_cell_measurement_temperature 296(2)

_cell_measurement_reflns_used ?

_cell_measurement_theta_min ?

_cell_measurement_theta_max ?

_exptl_crystal_description needle-shaped

_exptl_crystal_colour 'pale yellow'

_exptl_crystal_size_max 0.15

_exptl_crystal_size_mid 0.13

_exptl_crystal_size_min 0.09

_exptl_crystal_density_meas ?

_exptl_crystal_density_diffrn 1.279

_exptl_crystal_density_method 'not measured'

_exptl_crystal_F_000 644

_exptl_absorpt_coefficient_mu 0.085

_exptl_absorpt_correction_type empirical

_exptl_absorpt_correction_T_min 0.9449

_exptl_absorpt_correction_T_max 0.9748

_exptl_absorpt_process_details 'SADABAS 2.10 (Bruker 2003)'

_exptl_special_details

;

?

;

_diffrn_ambient_temperature 296(2)

_diffrn_radiation_wavelength 0.71073

_diffrn_radiation_type MoK\a

_diffrn_radiation_source 'fine-focus sealed tube'

_diffrn_radiation_monochromator graphite

_diffrn_measurement_device_type 'Bruker SMART APEX'

_diffrn_measurement_method /w-scans

_diffrn_detector_area_resol_mean ?

_diffrn_standards_number ?

_diffrn_standards_interval_count ?

_diffrn_standards_interval_time ?

_diffrn_standards_decay_% 0.5

_diffrn_reflns_number 26107

_diffrn_reflns_av_R_equivalents 0.0233

_diffrn_reflns_av_sigmaI/netI 0.0245

_diffrn_reflns_limit_h_min -12

_diffrn_reflns_limit_h_max 12

_diffrn_reflns_limit_k_min -16

_diffrn_reflns_limit_k_max 16

_diffrn_reflns_limit_l_min -19

_diffrn_reflns_limit_l_max 19

_diffrn_reflns_theta_min 1.44

_diffrn_reflns_theta_max 28.35

_reflns_number_total 7814

_reflns_number_gt 5517

_reflns_threshold_expression >2sigma(I)

_computing_data_collection 'SMART 5.628 (Bruker, 2003)'

_computing_cell_refinement 'SAINT 6.45 ( Bruker, 2003)'

_computing_data_reduction 'SAINT 6.45 (Bruker, 2003)'

_computing_structure_solution SIR-97

_computing_structure_refinement 'SHELXL-97 (Sheldrick, 1997)'

_computing_molecular_graphics 'Diamond 2.1e'

_computing_publication_material ?

_refine_special_details

;

Refinement of F^2^ against ALL reflections. The weighted R-factor wR and

goodness of fit S are based on F^2^, conventional R-factors R are based

on F, with F set to zero for negative F^2^. The threshold expression of

F^2^ > 2sigma(F^2^) is used only for calculating R-factors(gt) etc. and is

not relevant to the choice of reflections for refinement. R-factors based

on F^2^ are statistically about twice as large as those based on F, and R-

factors based on ALL data will be even larger.

;

_refine_ls_structure_factor_coef Fsqd

_refine_ls_matrix_type full

_refine_ls_weighting_scheme calc

_refine_ls_weighting_details

'calc w=1/[\s^2^(Fo^2^)+(0.1504P)^2^+0.7716P] where P=(Fo^2^+2Fc^2^)/3'

_atom_sites_solution_primary direct

_atom_sites_solution_secondary difmap

_atom_sites_solution_hydrogens geom

_refine_ls_hydrogen_treatment constr

_refine_ls_extinction_method none

_refine_ls_extinction_coef ?

_refine_ls_number_reflns 7814

_refine_ls_number_parameters 404

_refine_ls_number_restraints 1

_refine_ls_R_factor_all 0.1009

_refine_ls_R_factor_gt 0.0771

_refine_ls_wR_factor_ref 0.2682

_refine_ls_wR_factor_gt 0.2389

_refine_ls_goodness_of_fit_ref 1.040

_refine_ls_restrained_S_all 1.040

_refine_ls_shift/su_max 0.002

_refine_ls_shift/su_mean 0.000

loop_

_atom_site_label

_atom_site_type_symbol

_atom_site_fract_x

_atom_site_fract_y

_atom_site_fract_z

_atom_site_U_iso_or_equiv

_atom_site_adp_type

_atom_site_occupancy

_atom_site_symmetry_multiplicity

_atom_site_calc_flag

_atom_site_refinement_flags

_atom_site_disorder_assembly

_atom_site_disorder_group

N1 N 0.1202(3) 1.2670(2) -0.08416(18) 0.0657(6) Uani 1 1 d . . .

N2 N 0.5367(3) 0.56024(18) 0.22423(18) 0.0595(6) Uani 1 1 d . . .

N3 N 0.2961(2) 1.03675(16) 0.32417(13) 0.0419(4) Uani 1 1 d . . .

O3 O -0.0077(2) 0.83640(18) 0.44140(16) 0.0544(5) Uani 1 1 d . . .

H4N H -0.0919 0.8658 0.4636 0.065 Uiso 1 1 calc R . .

O1 O 0.3656(2) 0.93345(14) 0.07556(12) 0.0488(4) Uani 1 1 d . . .

O2 O 0.2962(2) 1.10124(18) 0.45603(13) 0.0633(5) Uani 1 1 d . . .

C1 C 0.4910(2) 0.8284(2) 0.30216(16) 0.0432(5) Uani 1 1 d . . .

H1 H 0.5070 0.8535 0.3531 0.052 Uiso 1 1 calc R . .

C2 C 0.5250(3) 0.7155(2) 0.30147(17) 0.0457(5) Uani 1 1 d . . .

H2 H 0.5619 0.6660 0.3519 0.055 Uiso 1 1 calc R . .

C3 C 0.5044(2) 0.67391(19) 0.22509(17) 0.0442(5) Uani 1 1 d . . .

C4 C 0.4498(3) 0.7523(2) 0.15098(17) 0.0453(5) Uani 1 1 d . . .

H4 H 0.4360 0.7284 0.0990 0.054 Uiso 1 1 calc R . .

C5 C 0.2520(3) 1.2022(2) 0.14363(19) 0.0531(6) Uani 1 1 d . . .

H5 H 0.2533 1.2379 0.1916 0.064 Uiso 1 1 calc R . .

C6 C 0.1835(3) 1.2594(2) 0.0695(2) 0.0575(6) Uani 1 1 d . . .

H6 H 0.1384 1.3317 0.0691 0.069 Uiso 1 1 calc R . .

C7 C 0.1806(3) 1.2100(2) -0.00597(18) 0.0503(6) Uani 1 1 d . . .

C8 C 0.2446(3) 1.0992(2) 0.00140(17) 0.0479(5) Uani 1 1 d . . .

H8 H 0.2424 1.0624 -0.0457 0.058 Uiso 1 1 calc R . .

C9 C 0.3942(2) 1.03077(18) 0.23242(15) 0.0398(5) Uani 1 1 d . . .

C10 C 0.4336(2) 0.90648(18) 0.22925(15) 0.0376(4) Uani 1 1 d . . .

C11 C 0.4159(2) 0.86536(19) 0.15395(15) 0.0395(5) Uani 1 1 d . . .

C12 C 0.3199(2) 1.09242(19) 0.14991(16) 0.0410(5) Uani 1 1 d . . .

C13 C 0.3111(2) 1.04319(19) 0.07772(16) 0.0415(5) Uani 1 1 d . . .

C14 C 0.5300(2) 1.08704(18) 0.23997(17) 0.0430(5) Uani 1 1 d . . .

C15 C 0.6613(3) 1.1073(2) 0.1768(2) 0.0555(6) Uani 1 1 d . . .

H15 H 0.6739 1.0920 0.1175 0.067 Uiso 1 1 calc R . .

C16 C 0.7745(3) 1.1511(2) 0.2034(3) 0.0669(8) Uani 1 1 d . . .

H16 H 0.8642 1.1651 0.1616 0.080 Uiso 1 1 calc R . .

C17 C 0.7556(3) 1.1741(2) 0.2918(3) 0.0677(8) Uani 1 1 d . . .

H17 H 0.8335 1.2018 0.3089 0.081 Uiso 1 1 calc R . .

C18 C 0.6222(3) 1.1564(2) 0.3548(2) 0.0592(7) Uani 1 1 d . . .

H18 H 0.6088 1.1731 0.4135 0.071 Uiso 1 1 calc R . .

C19 C 0.5092(3) 1.11285(19) 0.32744(18) 0.0453(5) Uani 1 1 d . . .

C20 C 0.3577(3) 1.08496(19) 0.37854(17) 0.0452(5) Uani 1 1 d . . .

C21 C 0.0455(5) 1.3800(3) -0.0914(3) 0.0915(12) Uani 1 1 d . . .

H21B H 0.0948 1.4222 -0.0605 0.139(19) Uiso 1 1 calc R . .

H21A H 0.0572 1.4201 -0.1579 0.15(2) Uiso 1 1 calc R . .

C22 C -0.1093(6) 1.3809(5) -0.0512(6) 0.147(2) Uani 1 1 d . . .

H22A H -0.1586 1.3348 -0.0779 0.220 Uiso 1 1 calc R . .

H22B H -0.1511 1.4579 -0.0649 0.220 Uiso 1 1 calc R . .

H22C H -0.1220 1.3505 0.0161 0.220 Uiso 1 1 calc R . .

C23 C 0.1220(3) 1.2125(3) -0.1612(2) 0.0648(7) Uani 1 1 d . . .

H23A H 0.2181 1.1730 -0.1737 0.078 Uiso 1 1 calc R . .

H23B H 0.1105 1.2715 -0.2180 0.078 Uiso 1 1 calc R . .

C24 C 0.0036(4) 1.1286(3) -0.1418(3) 0.0900(11) Uani 1 1 d . . .

H24A H 0.0096 1.0726 -0.0834 0.135 Uiso 1 1 calc R . .

H24B H 0.0177 1.0914 -0.1926 0.135 Uiso 1 1 calc R . .

H24C H -0.0921 1.1685 -0.1369 0.135 Uiso 1 1 calc R . .

C25 C 0.5257(6) 0.5223(5) 0.1410(4) 0.1171(16) Uiso 1 1 d D . .

H25A H 0.4583 0.5773 0.1078 0.140 Uiso 1 1 calc R . .

H25B H 0.6225 0.5304 0.1004 0.140 Uiso 1 1 calc R . .

C26 C 0.4857(11) 0.4240(9) 0.1449(7) 0.211(4) Uiso 1 1 d D . .

H26A H 0.4232 0.4306 0.0992 0.317 Uiso 1 1 calc R . .

H26B H 0.4319 0.3919 0.2072 0.317 Uiso 1 1 calc R . .

H26C H 0.5717 0.3752 0.1312 0.317 Uiso 1 1 calc R . .

C27 C 0.6031(3) 0.4822(2) 0.2992(2) 0.0594(7) Uani 1 1 d . . .

H27B H 0.6755 0.5221 0.3159 0.064(8) Uiso 1 1 calc R . .

H27A H 0.6553 0.4192 0.2754 0.067(9) Uiso 1 1 calc R . .

C28 C 0.4922(5) 0.4345(3) 0.3877(3) 0.0836(10) Uani 1 1 d . . .

H28A H 0.4363 0.4964 0.4101 0.125 Uiso 1 1 calc R . .

H28B H 0.5438 0.3887 0.4357 0.125 Uiso 1 1 calc R . .

H28C H 0.4260 0.3883 0.3733 0.125 Uiso 1 1 calc R . .

C29 C 0.1468(3) 0.9966(2) 0.34832(17) 0.0470(5) Uani 1 1 d . . .

H29A H 0.1248 0.9747 0.2946 0.056 Uiso 1 1 calc R . .

H29B H 0.0771 1.0589 0.3603 0.056 Uiso 1 1 calc R . .

C30 C 0.1244(3) 0.8953(2) 0.43467(18) 0.0501(6) Uani 1 1 d . . .

H30A H 0.2102 0.8420 0.4312 0.060 Uiso 1 1 calc R . .

H30B H 0.1170 0.9221 0.4914 0.060 Uiso 1 1 calc R . .

loop_

_atom_site_aniso_label

_atom_site_aniso_U_11

_atom_site_aniso_U_22

_atom_site_aniso_U_33

_atom_site_aniso_U_23

_atom_site_aniso_U_13

_atom_site_aniso_U_12

N1 0.0714(15) 0.0603(14) 0.0652(14) -0.0071(11) -0.0288(12) 0.0071(12)

N2 0.0768(15) 0.0436(11) 0.0677(14) -0.0239(10) -0.0291(12) 0.0110(10)

N3 0.0421(10) 0.0435(10) 0.0426(10) -0.0166(8) -0.0050(8) -0.0029(7)

O3 0.0464(11) 0.0502(11) 0.0661(13) -0.0210(10) 0.0008(9) -0.0055(9)

O1 0.0604(10) 0.0471(9) 0.0439(8) -0.0197(7) -0.0166(7) 0.0103(7)

O2 0.0653(12) 0.0773(13) 0.0568(11) -0.0384(10) -0.0086(9) 0.0043(10)

C1 0.0445(11) 0.0458(12) 0.0443(11) -0.0178(9) -0.0108(9) -0.0017(9)

C2 0.0464(12) 0.0450(12) 0.0485(12) -0.0143(10) -0.0139(10) 0.0017(9)

C3 0.0407(11) 0.0419(11) 0.0527(13) -0.0172(10) -0.0086(9) 0.0005(9)

C4 0.0452(12) 0.0485(12) 0.0488(12) -0.0230(10) -0.0118(9) 0.0024(9)

C5 0.0631(15) 0.0416(12) 0.0586(14) -0.0172(11) -0.0159(12) 0.0011(11)

C6 0.0634(16) 0.0426(12) 0.0666(16) -0.0128(11) -0.0175(13) 0.0054(11)

C7 0.0476(13) 0.0487(13) 0.0517(13) -0.0056(10) -0.0116(10) -0.0024(10)

C8 0.0487(13) 0.0518(13) 0.0440(12) -0.0138(10) -0.0093(10) 0.0005(10)

C9 0.0397(11) 0.0388(10) 0.0427(11) -0.0143(9) -0.0054(8) -0.0040(8)

C10 0.0362(10) 0.0386(10) 0.0402(10) -0.0139(8) -0.0055(8) -0.0038(8)

C11 0.0372(10) 0.0448(11) 0.0379(10) -0.0137(9) -0.0056(8) -0.0010(8)

C12 0.0426(11) 0.0395(11) 0.0416(11) -0.0111(9) -0.0071(9) -0.0034(8)

C13 0.0394(11) 0.0414(11) 0.0421(11) -0.0113(9) -0.0032(8) -0.0003(8)

C14 0.0431(11) 0.0344(10) 0.0547(13) -0.0155(9) -0.0105(9) -0.0021(8)

C15 0.0485(13) 0.0496(13) 0.0695(16) -0.0213(12) -0.0017(12) -0.0093(10)

C16 0.0468(14) 0.0505(14) 0.104(2) -0.0227(15) -0.0061(14) -0.0101(11)

C17 0.0523(15) 0.0521(15) 0.111(3) -0.0291(16) -0.0321(16) -0.0032(12)

C18 0.0639(16) 0.0491(14) 0.0780(18) -0.0281(13) -0.0311(14) 0.0038(12)

C19 0.0484(12) 0.0372(11) 0.0574(13) -0.0207(10) -0.0169(10) 0.0032(9)

C20 0.0517(13) 0.0421(11) 0.0474(12) -0.0206(10) -0.0139(10) 0.0068(9)

C21 0.118(3) 0.067(2) 0.094(3) -0.0108(19) -0.054(2) 0.021(2)

C22 0.101(4) 0.125(4) 0.222(7) -0.065(5) -0.035(4) 0.031(3)

C23 0.0638(17) 0.0722(18) 0.0522(15) -0.0002(13) -0.0170(13) -0.0035(14)

C24 0.087(3) 0.086(2) 0.103(3) -0.011(2) -0.044(2) -0.013(2)

C27 0.0623(16) 0.0486(14) 0.0732(18) -0.0238(13) -0.0223(13) 0.0123(12)

C28 0.103(3) 0.0605(18) 0.080(2) -0.0123(16) -0.008(2) -0.0017(18)

C29 0.0425(12) 0.0526(13) 0.0462(12) -0.0147(10) -0.0060(9) -0.0024(10)

C30 0.0512(13) 0.0497(13) 0.0512(13) -0.0150(10) -0.0083(10) -0.0057(10)

_geom_special_details

;

All esds (except the esd in the dihedral angle between two l.s. planes)

are estimated using the full covariance matrix. The cell esds are taken

into account individually in the estimation of esds in distances, angles

and torsion angles; correlations between esds in cell parameters are only

used when they are defined by crystal symmetry. An approximate (isotropic)

treatment of cell esds is used for estimating esds involving l.s. planes.

;

loop_

_geom_bond_atom_site_label_1

_geom_bond_atom_site_label_2

_geom_bond_distance

_geom_bond_site_symmetry_2

_geom_bond_publ_flag

N1 C7 1.379(3) . ?

N1 C21 1.457(4) . ?

N1 C23 1.464(4) . ?

N2 C3 1.384(3) . ?

N2 C27 1.455(3) . ?

N2 C25 1.455(6) . ?

N3 C20 1.352(3) . ?

N3 C29 1.453(3) . ?

N3 C9 1.492(3) . ?

O3 C31 1.357(3) . ?

O3 C30 1.450(3) . ?

O3 H4N 0.8600 . ?

O1 C11 1.376(3) . ?

O1 C13 1.380(3) . ?

O2 C20 1.234(3) . ?

C1 C2 1.376(3) . ?

C1 C10 1.394(3) . ?

C1 H1 0.9300 . ?

C2 C3 1.412(3) . ?

C2 H2 0.9300 . ?

C3 C4 1.396(3) . ?

C4 C11 1.386(3) . ?

C4 H4 0.9300 . ?

C5 C6 1.373(4) . ?

C5 C12 1.401(3) . ?

C5 H5 0.9300 . ?

C6 C7 1.412(4) . ?

C6 H6 0.9300 . ?

C7 C8 1.398(3) . ?

C8 C13 1.385(3) . ?

C8 H8 0.9300 . ?

C9 C12 1.513(3) . ?

C9 C14 1.526(3) . ?

C9 C10 1.526(3) . ?

C10 C11 1.385(3) . ?

C12 C13 1.379(3) . ?

C14 C15 1.375(3) . ?

C14 C19 1.390(3) . ?

C15 C16 1.388(4) . ?

C15 H15 0.9300 . ?

C16 C17 1.389(5) . ?

C16 H16 0.9300 . ?

C17 C18 1.386(5) . ?

C17 H17 0.9300 . ?

C18 C19 1.391(3) . ?

C18 H18 0.9300 . ?

C19 C20 1.475(3) . ?

C21 C22 1.432(7) . ?

C21 H21B 0.9700 . ?

C21 H21A 0.9700 . ?

C22 H22A 0.9600 . ?

C22 H22B 0.9600 . ?

C22 H22C 0.9600 . ?

C23 C24 1.504(5) . ?

C23 H23A 0.9700 . ?

C23 H23B 0.9700 . ?

C24 H24A 0.9600 . ?

C24 H24B 0.9600 . ?

C24 H24C 0.9600 . ?

C25 C26 1.270(10) . ?

C25 H25A 0.9700 . ?

C25 H25B 0.9700 . ?

C26 H26A 0.9600 . ?

C26 H26B 0.9600 . ?

C26 H26C 0.9600 . ?

C27 C28 1.514(5) . ?

C27 H27B 0.9700 . ?

C27 H27A 0.9700 . ?

C28 H28A 0.9600 . ?

C28 H28B 0.9600 . ?

C28 H28C 0.9600 . ?

C29 C30 1.523(4) . ?

C29 H29A 0.9700 . ?

C29 H29B 0.9700 . ?

C30 H30A 0.9700 . ?

C30 H30B 0.9700 . ?

loop_

_geom_angle_atom_site_label_1

_geom_angle_atom_site_label_2

_geom_angle_atom_site_label_3

_geom_angle

_geom_angle_site_symmetry_1

_geom_angle_site_symmetry_3

_geom_angle_publ_flag

C7 N1 C21 121.9(3) . . ?

C7 N1 C23 120.6(2) . . ?

C21 N1 C23 117.4(3) . . ?

C3 N2 C27 120.4(2) . . ?

C3 N2 C25 119.7(3) . . ?

C27 N2 C25 119.1(3) . . ?

C20 N3 C29 124.91(19) . . ?

C20 N3 C9 113.93(18) . . ?

C29 N3 C9 121.14(17) . . ?

C30 N4 H4N 118.0 . . ?

O4 N5 O3 122.6(3) . . ?

O4 N5 C34 118.7(3) . . ?

O3 N5 C34 118.7(3) . . ?

C11 O1 C13 118.39(17) . . ?

C2 C1 C10 122.3(2) . . ?

C2 C1 H1 118.8 . . ?

C10 C1 H1 118.8 . . ?

C1 C2 C3 120.8(2) . . ?

C1 C2 H2 119.6 . . ?

C3 C2 H2 119.6 . . ?

N2 C3 C4 121.7(2) . . ?

N2 C3 C2 121.3(2) . . ?

C4 C3 C2 117.1(2) . . ?

C11 C4 C3 120.8(2) . . ?

C11 C4 H4 119.6 . . ?

C3 C4 H4 119.6 . . ?

C6 C5 C12 122.7(2) . . ?

C6 C5 H5 118.7 . . ?

C12 C5 H5 118.7 . . ?

C5 C6 C7 121.0(2) . . ?

C5 C6 H6 119.5 . . ?

C7 C6 H6 119.5 . . ?

N1 C7 C8 120.7(2) . . ?

N1 C7 C6 122.8(2) . . ?

C8 C7 C6 116.5(2) . . ?

C13 C8 C7 121.1(2) . . ?

C13 C8 H8 119.4 . . ?

C7 C8 H8 119.4 . . ?

N3 C9 C12 110.72(17) . . ?

N3 C9 C14 100.41(17) . . ?

C12 C9 C14 114.65(18) . . ?

N3 C9 C10 110.36(17) . . ?

C12 C9 C10 110.01(17) . . ?

C14 C9 C10 110.34(17) . . ?

C11 C10 C1 116.45(19) . . ?

C11 C10 C9 122.14(19) . . ?

C1 C10 C9 121.40(18) . . ?

O1 C11 C10 122.89(19) . . ?

O1 C11 C4 114.56(19) . . ?

C10 C11 C4 122.5(2) . . ?

C13 C12 C5 115.9(2) . . ?

C13 C12 C9 122.32(19) . . ?

C5 C12 C9 121.8(2) . . ?

C12 C13 O1 123.2(2) . . ?

C12 C13 C8 122.8(2) . . ?

O1 C13 C8 113.93(19) . . ?

C15 C14 C19 121.0(2) . . ?

C15 C14 C9 129.2(2) . . ?

C19 C14 C9 109.8(2) . . ?

C14 C15 C16 118.4(3) . . ?

C14 C15 H15 120.8 . . ?

C16 C15 H15 120.8 . . ?

C15 C16 C17 120.8(3) . . ?

C15 C16 H16 119.6 . . ?

C17 C16 H16 119.6 . . ?

C18 C17 C16 120.9(3) . . ?

C18 C17 H17 119.5 . . ?

C16 C17 H17 119.5 . . ?

C17 C18 C19 117.9(3) . . ?

C17 C18 H18 121.1 . . ?

C19 C18 H18 121.1 . . ?

C14 C19 C18 120.9(2) . . ?

C14 C19 C20 108.84(19) . . ?

C18 C19 C20 130.2(2) . . ?

O2 C20 N3 125.3(2) . . ?

O2 C20 C19 127.8(2) . . ?

N3 C20 C19 106.88(19) . . ?

C22 C21 N1 115.3(4) . . ?

C22 C21 H21B 108.4 . . ?

N1 C21 H21B 108.4 . . ?

C22 C21 H21A 108.4 . . ?

N1 C21 H21A 108.4 . . ?

H21B C21 H21A 107.5 . . ?

C21 C22 H22A 109.5 . . ?

C21 C22 H22B 109.5 . . ?

H22A C22 H22B 109.5 . . ?

C21 C22 H22C 109.5 . . ?

H22A C22 H22C 109.5 . . ?

H22B C22 H22C 109.5 . . ?

N1 C23 C24 114.4(3) . . ?

N1 C23 H23A 108.7 . . ?

C24 C23 H23A 108.7 . . ?

N1 C23 H23B 108.7 . . ?

C24 C23 H23B 108.7 . . ?

H23A C23 H23B 107.6 . . ?

C23 C24 H24A 109.5 . . ?

C23 C24 H24B 109.5 . . ?

H24A C24 H24B 109.5 . . ?

C23 C24 H24C 109.5 . . ?

H24A C24 H24C 109.5 . . ?

H24B C24 H24C 109.5 . . ?

C26 C25 N2 123.5(7) . . ?

C26 C25 H25A 106.4 . . ?

N2 C25 H25A 106.4 . . ?

C26 C25 H25B 106.4 . . ?

N2 C25 H25B 106.4 . . ?

H25A C25 H25B 106.5 . . ?

C25 C26 H26A 109.5 . . ?

C25 C26 H26B 109.5 . . ?

H26A C26 H26B 109.5 . . ?

C25 C26 H26C 109.5 . . ?

H26A C26 H26C 109.5 . . ?

H26B C26 H26C 109.5 . . ?

N2 C27 C28 113.8(3) . . ?

N2 C27 H27B 108.8 . . ?

C28 C27 H27B 108.8 . . ?

N2 C27 H27A 108.8 . . ?

C28 C27 H27A 108.8 . . ?

H27B C27 H27A 107.7 . . ?

C27 C28 H28A 109.5 . . ?

C27 C28 H28B 109.5 . . ?

H28A C28 H28B 109.5 . . ?

C27 C28 H28C 109.5 . . ?

H28A C28 H28C 109.5 . . ?

H28B C28 H28C 109.5 . . ?

N3 C29 C30 113.4(2) . . ?

N3 C29 H29A 108.9 . . ?

C30 C29 H29A 108.9 . . ?

N3 C29 H29B 108.9 . . ?

C30 C29 H29B 108.9 . . ?

H29A C29 H29B 107.7 . . ?

N4 C30 C29 112.2(2) . . ?

N4 C30 H30A 109.2 . . ?

C29 C30 H30A 109.2 . . ?

N4 C30 H30B 109.2 . . ?

C29 C30 H30B 109.2 . . ?

H30A C30 H30B 107.9 . . ?

N4 C31 C36 119.1(2) . . ?

N4 C31 C32 122.5(2) . . ?

C36 C31 C32 118.4(2) . . ?

C33 C32 C31 120.0(2) . . ?

C33 C32 H32 120.0 . . ?

C31 C32 H32 120.0 . . ?

C32 C33 C34 120.7(2) . . ?

C32 C33 H33 119.6 . . ?

C34 C33 H33 119.6 . . ?

C33 C34 C35 120.4(2) . . ?

C33 C34 N5 120.2(3) . . ?

C35 C34 N5 119.4(3) . . ?

C36 C35 C34 119.5(2) . . ?

C36 C35 H35 120.2 . . ?

C34 C35 H35 120.2 . . ?

C35 C36 C31 120.9(2) . . ?

C35 C36 H36 119.6 . . ?

C31 C36 H36 119.6 . . ?

loop_

_geom_torsion_atom_site_label_1

_geom_torsion_atom_site_label_2

_geom_torsion_atom_site_label_3

_geom_torsion_atom_site_label_4

_geom_torsion

_geom_torsion_site_symmetry_1

_geom_torsion_site_symmetry_2

_geom_torsion_site_symmetry_3

_geom_torsion_site_symmetry_4

_geom_torsion_publ_flag

C10 C1 C2 C3 0.8(4) . . . . ?

C27 N2 C3 C4 175.7(2) . . . . ?

C25 N2 C3 C4 5.3(4) . . . . ?

C27 N2 C3 C2 -4.5(4) . . . . ?

C25 N2 C3 C2 -175.0(3) . . . . ?

C1 C2 C3 N2 -179.3(2) . . . . ?

C1 C2 C3 C4 0.5(3) . . . . ?

N2 C3 C4 C11 178.9(2) . . . . ?

C2 C3 C4 C11 -0.8(3) . . . . ?

C12 C5 C6 C7 -1.1(4) . . . . ?

C21 N1 C7 C8 176.5(3) . . . . ?

C23 N1 C7 C8 -0.1(4) . . . . ?

C21 N1 C7 C6 -4.3(5) . . . . ?

C23 N1 C7 C6 179.1(3) . . . . ?

C5 C6 C7 N1 -176.4(3) . . . . ?

C5 C6 C7 C8 2.8(4) . . . . ?

N1 C7 C8 C13 177.2(2) . . . . ?

C6 C7 C8 C13 -2.0(4) . . . . ?

C20 N3 C9 C12 -122.3(2) . . . . ?

C29 N3 C9 C12 55.9(3) . . . . ?

C20 N3 C9 C14 -0.7(2) . . . . ?

C29 N3 C9 C14 177.38(19) . . . . ?

C20 N3 C9 C10 115.7(2) . . . . ?

C29 N3 C9 C10 -66.2(3) . . . . ?

C2 C1 C10 C11 -1.7(3) . . . . ?

C2 C1 C10 C9 178.9(2) . . . . ?

N3 C9 C10 C11 128.9(2) . . . . ?

C12 C9 C10 C11 6.5(3) . . . . ?

C14 C9 C10 C11 -121.0(2) . . . . ?

N3 C9 C10 C1 -51.7(3) . . . . ?

C12 C9 C10 C1 -174.12(19) . . . . ?

C14 C9 C10 C1 58.4(3) . . . . ?

C13 O1 C11 C10 -9.2(3) . . . . ?

C13 O1 C11 C4 171.72(19) . . . . ?

C1 C10 C11 O1 -177.60(19) . . . . ?

C9 C10 C11 O1 1.8(3) . . . . ?

C1 C10 C11 C4 1.4(3) . . . . ?

C9 C10 C11 C4 -179.2(2) . . . . ?

C3 C4 C11 O1 178.9(2) . . . . ?

C3 C4 C11 C10 -0.1(3) . . . . ?

C6 C5 C12 C13 -1.5(4) . . . . ?

C6 C5 C12 C9 -179.7(2) . . . . ?

N3 C9 C12 C13 -130.3(2) . . . . ?

C14 C9 C12 C13 116.9(2) . . . . ?

C10 C9 C12 C13 -8.1(3) . . . . ?

N3 C9 C12 C5 47.8(3) . . . . ?

C14 C9 C12 C5 -64.9(3) . . . . ?

C10 C9 C12 C5 170.0(2) . . . . ?

C5 C12 C13 O1 -176.7(2) . . . . ?

C9 C12 C13 O1 1.6(3) . . . . ?

C5 C12 C13 C8 2.3(3) . . . . ?

C9 C12 C13 C8 -179.4(2) . . . . ?

C11 O1 C13 C12 7.6(3) . . . . ?

C11 O1 C13 C8 -171.5(2) . . . . ?

C7 C8 C13 C12 -0.6(4) . . . . ?

C7 C8 C13 O1 178.5(2) . . . . ?

N3 C9 C14 C15 179.8(2) . . . . ?

C12 C9 C14 C15 -61.5(3) . . . . ?

C10 C9 C14 C15 63.4(3) . . . . ?

N3 C9 C14 C19 3.3(2) . . . . ?

C12 C9 C14 C19 121.9(2) . . . . ?

C10 C9 C14 C19 -113.2(2) . . . . ?

C19 C14 C15 C16 1.9(4) . . . . ?

C9 C14 C15 C16 -174.3(2) . . . . ?

C14 C15 C16 C17 -0.1(4) . . . . ?

C15 C16 C17 C18 -1.4(4) . . . . ?

C16 C17 C18 C19 1.2(4) . . . . ?

C15 C14 C19 C18 -2.1(4) . . . . ?

C9 C14 C19 C18 174.8(2) . . . . ?

C15 C14 C19 C20 178.5(2) . . . . ?

C9 C14 C19 C20 -4.6(3) . . . . ?

C17 C18 C19 C14 0.6(4) . . . . ?

C17 C18 C19 C20 179.7(2) . . . . ?

C29 N3 C20 O2 0.2(4) . . . . ?

C9 N3 C20 O2 178.2(2) . . . . ?

C29 N3 C20 C19 -179.9(2) . . . . ?

C9 N3 C20 C19 -1.9(3) . . . . ?

C14 C19 C20 O2 -176.1(2) . . . . ?

C18 C19 C20 O2 4.7(4) . . . . ?

C14 C19 C20 N3 4.0(3) . . . . ?

C18 C19 C20 N3 -175.2(2) . . . . ?

C7 N1 C21 C22 -87.4(5) . . . . ?

C23 N1 C21 C22 89.3(5) . . . . ?

C7 N1 C23 C24 79.5(4) . . . . ?

C21 N1 C23 C24 -97.3(4) . . . . ?

C3 N2 C25 C26 -146.4(7) . . . . ?

C27 N2 C25 C26 43.0(9) . . . . ?

C3 N2 C27 C28 82.5(3) . . . . ?

C25 N2 C27 C28 -106.9(4) . . . . ?

C20 N3 C29 C30 -65.8(3) . . . . ?

C9 N3 C29 C30 116.3(2) . . . . ?

C31 N4 C30 C29 100.6(3) . . . . ?

N3 C29 C30 N4 -163.33(19) . . . . ?

C30 N4 C31 C36 164.3(2) . . . . ?

C30 N4 C31 C32 -14.6(4) . . . . ?

N4 C31 C32 C33 176.5(2) . . . . ?

C36 C31 C32 C33 -2.5(4) . . . . ?

C31 C32 C33 C34 -0.1(4) . . . . ?

C32 C33 C34 C35 1.7(4) . . . . ?

C32 C33 C34 N5 -176.8(3) . . . . ?

O4 N5 C34 C33 -2.7(4) . . . . ?

O3 N5 C34 C33 176.7(3) . . . . ?

O4 N5 C34 C35 178.8(3) . . . . ?

O3 N5 C34 C35 -1.8(4) . . . . ?

C33 C34 C35 C36 -0.6(4) . . . . ?

N5 C34 C35 C36 177.8(2) . . . . ?

C34 C35 C36 C31 -2.0(4) . . . . ?

N4 C31 C36 C35 -175.5(2) . . . . ?

C32 C31 C36 C35 3.5(4) . . . . ?

_diffrn_measured_fraction_theta_max 0.993

_diffrn_reflns_theta_full 28.35

_diffrn_measured_fraction_theta_full 0.993

_refine_diff_density_max 0.624

_refine_diff_density_min -0.355

_refine_diff_density_rms 0.059
